# Limited Amount of Formula May Facilitate Breastfeeding: Randomized, Controlled Trial to Compare Standard Clinical Practice versus Limited Supplemental Feeding

**DOI:** 10.1371/journal.pone.0150053

**Published:** 2016-02-26

**Authors:** Zbyněk Straňák, Simona Feyereislova, Marcela Černá, Jana Kollárová, Jaroslav Feyereisl

**Affiliations:** 1 Third Faculty of Medicine, Charles University, Prague, Czech Republic; 2 Department of Neonatology, Institute for the Care of Mother and Child, Prague, Czech Republic; 3 Department of Obstetrics and Gynecology, Institute for the Care of Mother and Child, Prague, Czech Republic; Emory University School of Medicine, UNITED STATES

## Abstract

**Objectives:**

Breastfeeding is known to reduce infant morbidity and improve well-being. Nevertheless, breastfeeding rates remain low despite public health efforts. Our study aims to investigate the effect of controlled limited formula usage during birth hospitalisation on breastfeeding, using the primary hypothesis that early limited formula feeds in infants with early weight loss will not adversely affect the rate of exclusive or any breastfeeding as measured at discharge, 3 and 6 months of age.

**Material and Methods:**

We randomly assigned 104 healthy term infants, 24 to 48 hours old, with ≥ 5% loss of birth weight to controlled limited formula (CLF) intervention (10 ml formula by syringe after each breastfeeding, discontinued at onset of lactation) or control group (standard approach, SA). Groups were compared for demographic data and breastfeeding rates at discharge, 3 months and 6 months of age (p-values adjusted for multiple testing).

**Results:**

Fifty newborns were analysed in CLF and 50 in SA group. There were no differences in demographic data or clinical characteristics between groups. We found no evidence of difference between treatment groups in the rates of exclusive as well as any breastfeeding at discharge (p-value 0.2 and >0.99 respectively), 3 months (p-value 0.12 and 0.10) and 6 months of infants’ age (p-value 0.45 and 0.34 respectively). The percentage weight loss during hospitalisation was significantly higher in the SA group (7.3% in CLF group, 8.4% in SA group, p = 0.002).

**Conclusion:**

The study shows that controlled limited formula use does not have an adverse effect on rates of breastfeeding in the short and long term. Larger studies are needed to confirm a possible potential in controlled limited formula use to support establishing breastfeeding and to help to improve the rates of breastfeeding overall.

**Trial Registration:**

ISRCTN registry ISRCTN61915183

## Introduction

Breastfeeding is considered to be the best and most cost-effective intervention in newborns in order to reduce morbidity, improve growth and short-term as well as long-term wellbeing. The properties of human breast milk help to facilitate developmental changes during critical periods of brain, immune system or gut development. Major organisations, including the World Health Organisation and UNICEF, recommend exclusive breastfeeding for the first 6 months and continued breastfeeding for 2 years or more with adequate complementary feeding [1], as longer duration of breastfeeding has been shown to be associated with greater health benefits [2].

Although the benefits of breastfeeding are widely acknowledged, maintaining exclusive breastfeeding in longer-term remains to be a challenge. WHO states that globally less than 40% of infants under six months of age are exclusively breastfed [3]. National Center for Chronic Disease Prevention and Health Promotion stated that in 2011 although 79% of newborn infants started to breastfeed, only 49% were breastfeeding at 6 months and 27% at 12 months of age in the USA [4]. In Czech Republic (place of the study) the rate for exclusive breastfeeding is even lower and recent analysis revealed no differences in breastfeeding at discharge between Baby Friendly Initiative Hospitals and other hospitals. The Baby-Friendly Hospital Initiative is a worldwide programme launched by WHO and UNICEF in 1991. It is a global effort to implement practices that protect, promote and support breastfeeding and to ensure that all maternities, whether free standing or in a hospital, become centers of breastfeeding support by complying with specifically developed implementation guides and global criteria including 10 specific steps to support successful breastfeeding [5,6].

To enable mothers to establish and sustain exclusive breastfeeding, WHO and UNICEF suggest initiation of breastfeeding within the first hour of life; breastfeeding without any additional food or drink, not even water; breastfeeding on demand as well as avoidance of bottles, teats and pacifiers [7]. In general public health efforts today tend to emphasise reduction in the use of formula during the birth hospitalisation in order to improve breastfeeding rates and duration. Some published literature suggests additional formula feeds during birth hospitalisation may be associated with earlier discontinuation of breastfeeding [8,9], others however have shown no benefit to duration of breastfeeding from formula restricting policy during birth hospitalisation [10]. Flaherman et al. performed a randomised controlled trial assigning 40 term infants, 24 to 48 hours old, with weight loss of ≥ 5% of birth weight to early limited formula (ELF) intervention (10 mL formula by syringe after each breastfeeding and discontinued when mature milk production began) or control (continued exclusive breastfeeding). The study showed, that early limited formula may reduce longer-term formula use at 1 week and increase breastfeeding at 3 months for some infants [11].

A study focusing on the problems experienced by breastfeeding mothers shows that one of the most common reasons cited by mothers who opt to stop breastfeeding in early neonatal period is anxiety over insufficient milk supply [12]. The physiological process of onset of lactation does not usually occur immediately after delivery; instead it may take up to several days for mature milk production to settle in. It is the relatively small volumes of colostrum and the unsettled behaviour of the newborn prone to dissatisfaction during these first days of life that, despite offered reassurance, may be misinterpreted by the mother as insufficient milk supply and lead to early cessation of breastfeeding. Approaches to this problem vary widely. Although some, including The Baby Friendly Hospital Initiative promote elimination of supplementary top-up formula feeds for healthy infants [13], others perceive benefits from early supplementation of other fluids or foods. Optimizing clinical practice regarding establishing breastfeeding and formula use during early neonatal period may prove to have a major impact on overall health benefit for newborn infants, if this helps to prolong overall breastfeeding duration and raise the numbers of ideally exclusively breastfed infants up to 6 months of age.

In this study, we aim to investigate the role and effect of limited formula use on breastfeeding and its discontinuation. The primary outcome is to investigate the rates of exclusive as well as any breastfeeding at 3 months of age. We hypothesize, that early limited formula feeds in infants with early weight loss will not adversely affect the rates of exclusive or any breastfeeding in short-term and long-term. Infants who lose 5% or more of their birth weight during the first 48 hours after delivery are known to be at higher risk of excessive weight loss and hence are at higher risk of eventual formula supplementation as well as the risk of maternal concern regarding insufficient milk supply with the feared result of shorter breastfeeding duration. These at risk newborns may hence benefit from an early limited formula use, where the given controlled amounts for a limited time would not be expected to interfere with breastfeeding as recommended.

## Materials and Methods

The study was conducted at the Institute for the Care of Mother and Child in Prague. Patients were recruited from the 1^st^ of April 2014 until the 29^th^ of August 2014. Follow up was completed on the 27^th^ of February 2015. The trial was registered in the ISRCTN registry with the ID number ISRCTN61915183. As trial registration is not required in advance of performing a study that has been approved by the ethics committee in Czech Republic, patient recruitment was commenced before the trial was registered for the purpose of publication. The authors confirm that all ongoing and related trials for this intervention are registered.

Infants were eligible for randomisation when their weight loss was ≥ 5 per cent between 24^th^ and 48^th^ hour of life. Only healthy, singleton, appropriate for gestational age (AGA) term neonates, born after uncomplicated pregnancy and delivery, who had no severe congenital defects were enrolled. Mothers of the included infants were planning to breastfeed for a long time and all were Czech citizens. Mothers with serious complications (hypertension, diabetes, systemic diseases, drug abuse) or using therapy that might affect breastfeeding (antidepressants) were excluded. Written informed consent was obtained from all mothers by a study doctor. This study, including the consent procedure, was approved by the Local Ethics Committee and Local Committee on Human Research on the 19^th^ of December 2013 (The Ethics Committee on Clinical Trial on Human Medicinal Products, Prague 4).

To estimate the sample size, data from the randomized controlled trial published by Flaherman et al. [11] were used instead of performing a preliminary study. For the given effect size (population proportions of 0.95 versus 0.75) and alpha (0.05 2-tailed) with power of 0.80, the estimated sample sizes are 49 in each group. This means that 80% of studies would be expected to yield a significant effect, rejecting the null hypothesis that the two population proportions are equal. The sample size estimation was performed using the software SamplePower 3.

Using the sealed envelope technique and randomization with permuted blocks, we randomly assigned 104 mother-infant pairs to either controlled limited formula group (CLF) or standard approach group (SA). The randomization sequence was generated and the sealed envelopes in blocks of 8 were prepared by the hospitals’ administration office staff.

All mothers irrespective of assigned study group were educated by a specialised nurse regarding breastfeeding. As part of the hospitals’ standard service, all to be mothers are offered optional participation in antenatal educational courses of 2 hours duration and are informed in antenatal clinics regarding the possibility of obtaining information about breastfeeding from the hospital website, available printed leaflets and rentable short movie. In addition all mothers take part in a general breastfeeding session of 60 minutes duration on the ward during the birth hospitalisation, participation in this session was made mandatory for all mothers participating in the study. Specialised nurses supervise all breastfeeding mothers regarding breastfeeding techniques on the wards until breastfeeding is established as a routine hospital practice and additional help was available on mothers’ request. All participating mothers were asked about the used sources of breastfeeding information and the response noted in the case report form (not analysed for the purpose of the study).

Infants in the controlled limited formula group (CLF—intervention group) were given a set volume of 10ml of formula (HIPP NE, HIPP Inc., Germany) after each breastfeed until adequate milk production began. Infants in the standard approach group (SA—control group) were exclusively breastfed. Supplemental feeds were administered only in indicated cases. This included excessive weight loss (more than 10% of birth weight), irritability of the newborn, in terms of unsettling cry and hungry behaviour, and on mothers specific request. Infants in the control group, if requiring supplemental feeding, were given breastmilk from the breastmilk-bank or formula according to the mothers’ choice. All formula feeds were always given using the syringe-technique.

The primary outcome measures were the rates of any breastfeeding and of exclusive breastfeeding at 3 months of the infants´ age. The secondary outcome measures included the rates of any breastfeeding and exclusive breastfeeding at the time of hospital discharge and at 6 months of the infants´ age, as well as the percentage weight loss during birth hospitalisation. Clinical data for all infants involved, including any complications occurring during the hospitalisation, were collected prospectively and recorded using a case report form. Using transcutaneous measurement of bilirubin by Icterometer (Minolta Air Shields Jaundice Meter JM-103), all participants have been regularly checked for hyperbilirubinaemia as according to standard hospital guidelines. Levels of bilirubin above 200 umol/l have been recorded, as per standard hospital guidelines levels of serum-bilirubin were measured and all cases requiring phototherapy noted. A research nurse who was blinded to group allocation assessed outcomes during a personal interview at discharge and by a telephone-interview at 3 and 6 months of the infants’ age. Breastfeeding was assessed on questioning the mother, firstly on exclusive breastfeeding and secondly on any breastfeeding with closed questions and answers recorded as yes or no.

Data was analysed using descriptive statistic methods. We used the Chi-Square Test of independence, or Fisher’s Exact Test for comparison of groups of categorised variables and the Mann-Whitney test for comparison of numerical variables. All p-values are calculated at level of significance of 0.05 (alpha = 0.05) and adjusted for multiple testing using the Bonferroni correction when appropriate. Statistical analysis was performed using the software IBM SPSS Statistics 22.0.0.1.

## Results

### Participant characteristics

**Fig 1:**
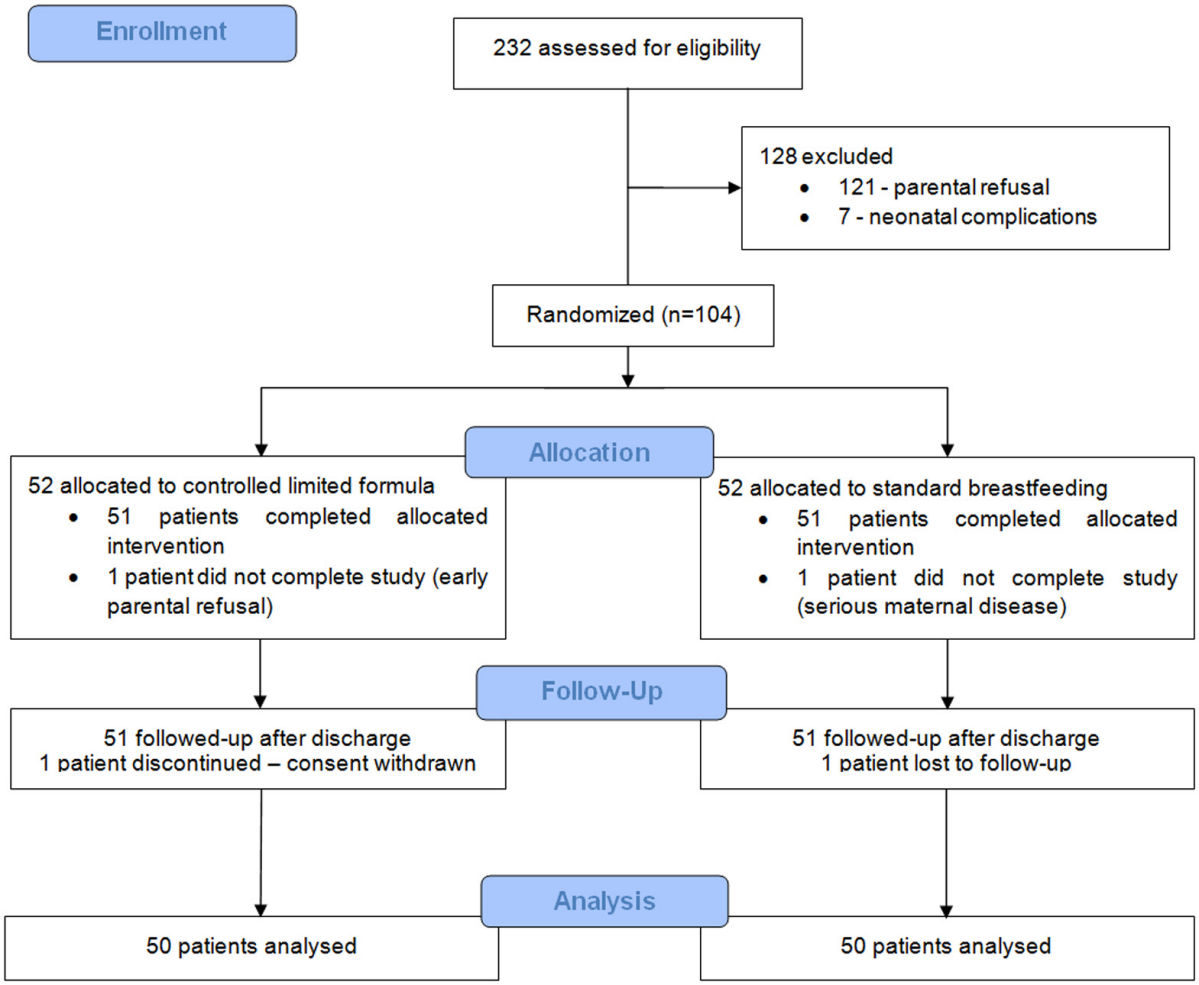
Flow chart of the randomized study.

Overall 52 (50%) infants were assigned to controlled limited formula group (CLF) and 52 (50%) to standard approach group (SA). Two participants from each group failed to complete the study, 50 participants in each group were analysed. Fig 1 shows the numbers of mother-infant pairs at enrolment, allocation, follow-up and for analysis.

All mothers involved in the study were planning and wishing to breastfeed over long term. Thematernal demographic parameters and general clinical characteristics of participating infants in each of the intervention groups are shown in Table 1.

**Table 1:** Demographic and Clinical Characteristics of the Study Groups.

	Controlled and limited formula (Intervention group, n = 50)	Standard approach (Control group, n = 50)	P value
Gestational age,wk, mean ± SD	39.3 ± 1.0	39.4 ± 1.1	0.35
Birth weight,g, mean ± SD	3367 ± 305	3324 ± 307	0.52
Weight at enrolment,g, mean ± SD	3158 ± 282	3113 ± 288	0.42
Infants‘ age at enrolment,hours, mean ± SD (95% CI)	31.3 ± 6.1 (CI 29.6–33.0)	32.0 ± 5.6 (CI 30.4–33.6)	0.35
Length of hospitalisation,days, mean ± SD (95% CI)	4 ± 0.7 (3.8–4.2)	4.4 ± 1.0 (4.1–4.7)	0.11
Number of breastfeeding attempts*, mean ± SD (95% CI)	21.7 ± 7.0 (19.8–23.7)	26.5 ± 9.5 (23.8–29.2)	0.01
Number of breastfeeding attempts normalized to lenght of stay, mean ± SD (95% CI)	8.2 ± 1.8 (7.6–8.7)	8.9 ± 1.8 (8.4–9.4)	0.02
Transcutaneous icterometry > 200 umol/l, n (%)	8 (16%)	18 (36%)	0.02
Maternal age, years, mean ± SD	31.6 ± 4.2	32.1 ± 4.0	0.47
Primiparous, n (%)	27 (54)	27 (54)	>0.99
Mode of delivery, n (%)			0.72
Vaginal delivery	32 (64)	30 (60)	
Acute Cesarean section	5 (10)	7 (14)	
Elective Cesarean section	13 (26)	13 (26)	
Mother with high school or more education, n (%)	47 (94)	48 (96)	0.84
Skin-to-skin at delivery room, n (%)	29 (58)	30 (60)	0.84
Plan to breastfeed, n (%)	50 (100%)	50 (100%)	>0.99

Abbreviations:

* = Number of breastfeeding attempts during hospitalisation (i.e. between enrolment and discharge)

### Delivery of the intervention and control

The intervention of controlled limited formula was delivered to 50 infants in the intervention group. Only 11 out of 50 (22%) infants in the control group were exclusively breastfed during hospitalisation. Supplemental feeding was needed in 39 cases (78%) within the control group. Out of these cases 31 (80%) were given breastmilk from the breastmilk-bank and 8 (20%) were given formula on specific parental request. In 10 cases within the control group, supplemental feeds were given from medical indication of excessive weight loss of ≥10% of birth weight during the hospitalisation, in all of the remaining cases in the control group, supplemental feeds were given on repeated and explicit parental demand. The maximum total volume of supplemental feeds given was 400ml in the CLF group and 475ml in the control group. The median amount of total volume of supplemental feeds given was 60 ml (interquartile range of 56) in the CLF group and 20ml (interquartile range of 90) in the control group. (See Table 2.)

**Table 2:** Comparison of supplemental feeds given by intervention group.

	Controlled and limited formula (Intervention group, n = 50)	Standard approach (Control group, n = 50)
Volume of supplemental feeds given overall, ml		
median	60	20
1^st^ quartile	40	2,5
3^rd^ quartile	96	92,5
Interquartile range	56	90
Maximum total volume of supplemental feeds given, ml	400	475

### Rates of breastfeeding

Our analysis showed no evidence of a difference in rates of exclusive breastfeeding as well as that of any breastfeeding at any time point among participants allocated to the intervention and control groups. The percentage of breastfeeding mothers however tends to be higher among those of the CLF group compared to the control group in both exclusive and any breastfeeding, particularly at 3 and 6 months of the infants’ age although this is not statistically significant and the percentage of mothers with any breastfeeding at discharge are very similar. The data are summarised in Table 3. The number of times a mother has attempted to breastfeed during hospitalisation (between enrolment and discharge) is higher in the control group compared to CLF group (p-value 0.01, shown in Table 1).

**Table 3:** Breastfeeding rates by Study Group. P-values adjusted for multiple testing using Bonferroni correction.

	Controlled and limited formula (Intervention group, n = 50)	Standard approach (Control group, n = 50)	P-value	Adjusted p-value	Odds ratio	95% Confidence Interval
Exclusive breastfeeding at discharge, n (%)	49 (98)	44 (88)	0.11	0.22	6.68	0.77–57.69
Breastfeeding at discharge, n (%)	50 (100)	49 (98)	>0.99	>0.99	Not applicable	Not applicable
Exclusive breastfeeding at 3 month, n (%)	42 (84)	34 (68)	0.06	0.12	2.47	0.94–6.46
Breastfeeding at 3 month, n (%)	46 (92)	39 (78)	0.05	0.10	3.24	0.96–11.00
Exclusive breastfeeding at 6 month, n (%)	32 (64)	26 (52)	0.22	0.45	1.64	0.74–3.66
Breastfeeding at 6 month, n (%)	40 (80)	34 (68)	0.17	0.34	1.88	0.76–4.69

The rates of exclusive breastfeeding at discharge, 3 and 6 months of age were not affected by the mode of delivery (vaginal versus cesarean section) or presence/absence of skin to skin contact in delivery room. No evidence of difference was shown when looking at the rates of exclusive breastfeeding at discharge, 3 and 6 months of infants’ age irrespective of treatment group as well as when analysis was performed only at 3 months (primary outcome) adjusted for treatment arm. These results are summarised in Tables 4 and 5.

**Table 4:** Comparison of the rates of exclusive breastfeeding in accordance to mode of delivery and skin-to-skin contact irrespective to patient group allocation.

	Mode of delivery			Skin to skin contact		
Rate of exclusive breastfeeding	Vaginal	Caesarean section	p-value	YES	NO	p-value
at discharge (%)	93.5	92.1	0.61	93.2	92.7	0.63
at 3 months (%)	77.4	73.7	0.7	79.7	70.7	0.54
at 6 months (%)	56.5	60.5	0.49	57.6	58.5	0.63

**Table 5:** Comparison of rates of exclusive breastfeeding in accordance to mode of delivery and skin-to-skin contact, adjusted for treatment arm.

	Mode of delivery			Skin to skin contact		
Rate of exclusive breastfeeding	Vaginal	Caesarean section		YES	NO	
CLF group (%)	64.3	35.7		60	40	
Control group (%)	61.8	38.2		65	35	
p-value			0.82			0.64

### Weight loss in participating newborn infants

**Fig 2:**
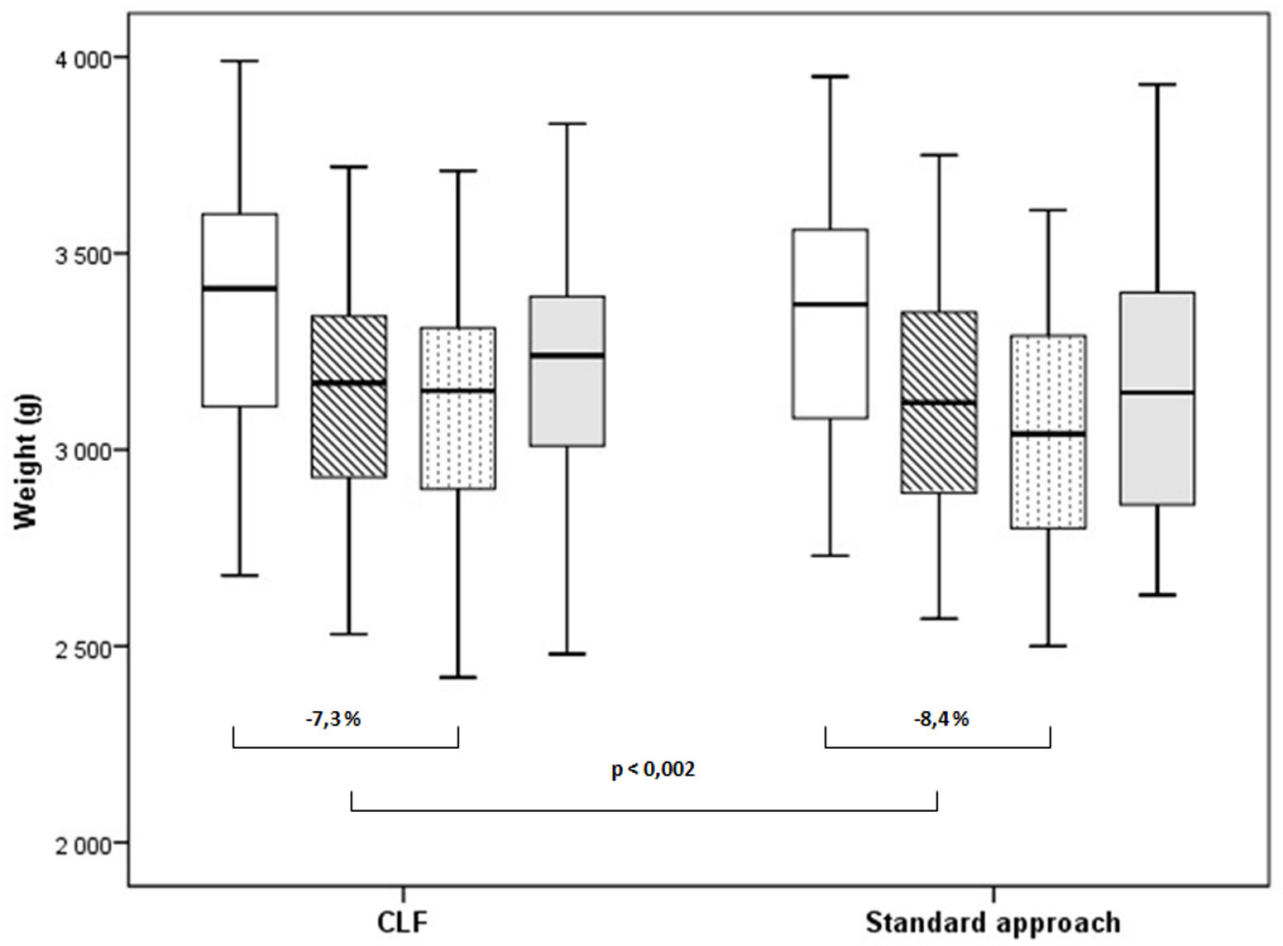
Differences in weight loss by intervention group. Median, first and third quartiles are shown, as well as the minimal and maximal values for birth weight—white box, weight at enrollment—striped box, lowest weight—dotted box, weight at discharge—grey box. Statistically significant difference in weight loss between groups is shown.

Although there was no statistically significant difference in birth weights or weights at enrolment between the intervention groups, there was a significant difference in the maximal body weight loss (expressed in percent—minus 7.3% in CLF group and minus 8.4% in control group respectively, p = 0.002). At the point of study enrolment, there were no infants with weight loss ≥10% of their birth weight, however eleven cohort infants lost ≥10% of their birth weight during the study, including 1 (2%) of 50 in the CLF group and 10 (20%) of 50 in the control group (p = 0.004). The comparison of weight changes are shown in Fig 2.

### Complications

Llevels of bilirubin above 200 umol/l were found in 8 cases (16%) in the CLF group and in 18 cases (36%) in the control group. This difference is statistically significant (p-value 0.02), Only 1 of all the cases, a participant from the control group, required phototherapy treatment (lasting 48 hours overall). There were no events of hypoglycaemia, gastrointestinal problems including feeds intolerance, sepsis, or diagnosis of neuromuscular disorders, inborn errors of metabolism and birth defects noted in any of the participating infants. No complications affecting ability to breastfeed were reported by any of the participating mothers.

## Discussion

Our study compared the use of controlled limited supplemental formula feeding with the standard clinical practice regarding the effect on initiation and maintenance of breastfeeding. Many hospitals, as opposed to the aim of Baby Friendly Hospitals Initiative, do use uncontrolled amounts of supplemental feeding [14] as part of their standard practice, as it is often asked for by parents. This approach tends to be linked to effectivity of breastfeeding. In contrast to some existing literature discouraging from the use of supplemental formula feeding during birth hospitalisation [8,9], our results show no evidence of difference in the rates of exclusive as well as any breastfeeding between participants allocated to the intervention of controlled and limited formula use as compared to those allocated to the control group. The study supports our hypothesis that supplemental controlled and limited formula feeding of at risk infants with weight loss of ≥ 5% birth weight does not adversely affect the rates of exclusive and any breastfeeding in short and long term. There seems to be a tendency shown by the slightly higher percentage of breastfeeding mothers in the CLF group at 3 and 6 months of the infants‘ age, which may suggest that controlled limited formula use during birth hospitalisation may actually support the onset of lactation and improve maintenance of breastfeeding in longer term in these cases, however due to lack of statistical significance, this needs to be confirmed in a larger study.

In this study, supplemental feedings were strictly defined in terms of amounts (these were chosen so that the newborns´ demand for breastfeeding would be maintained and would not interfere with the recommended [13] 8 to 12 breastfeeding times per day), form (always using the syringe to avoid nipple confusion that may be associated with bottle feeding) and duration (controlled limited formula feeding was terminated as soon as breastfeeding was established). This approach differs from some clinical practices of unstructured formula supplementation. Another way the controlled formula supplementaion may support and improve breastfeeding is the fact that it possibly alleviates maternal concerns regarding insufficient milk production. This is known to be one of the common reasons for breastfeeding discontinuation [12]. As shown, infants of the CLF group proved to have significantly lower maximal bodyweight loss and significantly lower rates of excessive weight loss (≥10%) as compared to the control group. By improving the infants’ weight and possibly hydration status, the behavioural pattern in terms of newborns dissatisfaction might also be improved, hence supporting the mother in her intention to continue breastfeeding whilst awaiting onset of mature milk production. In addition as formula usage after onset of lactation is associated with possible reduction in lactation and early cessation of breastfeeding [9] and knowing that infants with greater weight loss are at higher risk of their mothers opting to continue formula supplementation after breastfeeding is established [12], giving early controlled formula amounts for limited time may help to reduce the actual use of formula in longer term and hence improve the rates of breastfeeding or even exclusive breastfeeding at later time point. In the authors’ opinion, the general pressure on excluding supplemental formula feeding from birth hospitalisation, whilst being relevant with respect to some clinical approaches, may unintentionally potentially lower the chances of breastfeeding for some at risk cohorts that could be possibly identified and helped with the strategy of controlled limited formula feeding.

According to the available data on breastfeeding rates within Czech Republic, the overall rate of exclusive breastfeeding at 3 months of age is 33% and that of breastfeeding to some extent at 3 months is 62% [15]. Both of the groups observed in this study (CLF and SA) have achieved higher breastfeeding rates than the national average (84% and 68% respectively). Moreover, comparison with published data of the Baby Friendly Hospitals Initiative within Czech Republic, where the breastfeeding rates achieved at discharge are given as 86% [16], the breastfeeding rates at discharge in the CLF group are higher (98%), whereas those of the control group, i. e. the standard clinical approach (88%) are comparable.

Interestingly, in our cohort no statistically significant difference for breastfeeding at discharge and 3 or 6 months of age was found with respect to skin-to-skin contact occurance in delivery room and with respect to mode of delivery (vaginal or cesarean section). These, although being valid recommendations to help establish and maintain exclusive breastfeeding, do not seem to be the only determinants of breastfeeding success.

The fact that the number of breastfeeding times/attempts is significantly higher in the control group (p-value 0.02) even when normalized to length of stay, may be due to increased maternal effort to exclusively breastfeed. The significantly higher number of newborns with jaundice in control group (hyperbilirubinaemia of >200 umol/l as measured by transcutaneous icterometer, p-value 0.02) corresponds with published data on hyperbilirubinaemia in exclusively breastfed infants [17].

The limitations of our study include mainly the low rate of exclusively breastfed newborn infants for the entire duration of birth hospitalisation. Overall 78% of infants in the control group were given a supplemental feed at some point during the hospitalisation. Considering the inclusion criteria of the study (weight loss ≥ 5% of birth weight at enrolment), this may be underlining the need for a strategy alleviating maternal concerns, as in this group supplemental feeding, if not indicated medically (excessive weight loss, signs of dehydration), was only given at mothers explicit wish and all mothers were equally motivated to exclusively breastfeed at the time of enrolment. Comparing the given volumes of supplemental feeds in the two groups, the median of the control group is much lower than that in the CLF group, however the interquartile range (90 in control group compared to 56 in CLF group) shows the greater variety of the given amounts of supplemental feeds, in some cases comprising of larger amounts than the median amount given in the CLF group. This is due to the presence of few individuals in need of large amounts of supplemental feeds who may have skewed the distribution within the group. The relatively large number of newborns given supplemental feeds within the control group also limits the possibility for comparison with the CLF group, as the comparison mainly comprises controlled and limited versus uncontrolled formula feeding rather than formula feeding versus exclusive breastfeeding. This practical limitation of the study method with inability of prohibiting supplemental feeds in standard hospital practice might have contributed to the fact that differences in the breastfeeding rates have not been statistically significant and could have been expected to be greater in values. Another published study of similar design, comparing exclusive breastfeeding with early limited formula supplementation during birth hospitalisation in healthy term newborns with ≥5% birth weight loss has shown statistically signifacnt difference at 3 months in favour of the early limited formula feeding. In this randomized controlled study 15 (79%) of 19 infants assigned to the intervention group during the birth hospitalisation were breastfeeding exclusively, compared with 8 (42%) of 19 controls (P = 0.02) at 3 months of the infants‘ age [11]. In order to minimise possible influence of the given supplementational feeds, most infants in the control group were offered breast milk from breastmilk-bank (80% of all supplementational feeds in the control group), unless the parents wished explicitly for formula.

Another study limitation is posed by the highly selected cohort of mother-newborn pairs. The inclusion criteria exclude a large population of newborn infants (e.g. pre-term, small or large for gestational age) that might also benefit from improval of breastfeeding rates, however supplemental feeding and perhaps further supportive nutritional measures that may have to be taken due to many different reasons, make comparison difficult. In addition, the inclusion criteria of ≥ 5% body weight loss contributes to select specifically at risk infants, where foreseeable problems arise especially for exclusive breastfeeding throughout birth hospitalisation and hence mainly for the control group. This might have an effect on the achieved breastfeeding rates within this group, with a trend to lower breastfeeding rates than in the CLF group. The mothers involved in our study also possibly represent only a selected part of population. The recruitment of participating mother-infant pairs took place within one hospital only. There are relatively large regional differences with respect to education, income and invariably overall breastfeeding rates as well as numbers of mothers with the intention to exclusively breastfeed for longer term, all of which can affect the results of our comparison. Hence further research would be needed to confirm our results in larger sample size as well as in a population with greater diversity and better representation of regional differences.

In conclusion, the study shows that controlled limited formula use does not have an adverse effect on rates of breastfeeding in short and long term and may support establishing breastfeeding, as well as potentially help to improve the rates of breastfeeding overall.

## Supporting Information

S1 Table CONSORT checklist. (DOC)

S2 Table Data set. (Raw data set of the study.)
(DOCX)

S1 Text Study protocol original. (PDF)

S2 Text Case Report Form. (Original document in Czech language.)
(PDF)
