# Supplementary material for: Limited Amount of Formula May Facilitate Breastfeeding: Randomized, Controlled Trial to Compare Standard Clinical Practice versus Limited Supplemental Feeding
Source: PLoS One. 2016 Feb 26;11(2):e0150053. doi: 10.1371/journal.pone.0150053 (PMC4769147; doi:10.1371/journal.pone.0150053)
Supplement: S2 Table — (Raw data set of the study.) (DOCX) [file pone.0150053.s002.docx]

|  |  |  |  |  | Breastfeeding | | |  |  |  |  |
| --- | --- | --- | --- | --- | --- | --- | --- | --- | --- | --- | --- |
| Intervention | BW | Weight at discharge | Weight at enrollment | Lowest weight | discharge | 3M | 6M | TKImax | Mode of delivery | Skin to skin | Maternal age |
| CLF | 2 680 | 2 480 | -5,60 | 2 420 | 1 | 1 | 1 | 226 | 4 | 2 | 33 |
| Control | 2 730 | 2 630 | -5,86 | 2 500 | 1 | 1 | 1 | 165 | 4 | 2 | 34 |
| CLF | 2 740 | 2 530 | -5,84 | 2 520 | 1 | 1 | 1 | 199 | 1 | 1 | 37 |
| Control | 2 830 | 2 800 | -5,30 | 2 630 | 1 | 1 | 1 | 130 | 3 | 2 | 34 |
| CLF | 2 860 | 2 710 | -5,42 | 2 680 | 1 | 3 | 3 | 190 | 1 | 1 | 20 |
| Control | 2 890 | 2 670 | -5,54 | 2 650 | 1 | 1 | 1 | 215 | 4 | 2 | 32 |
| CLF | 2 890 | 2 760 | -7,27 | 2 680 | 1 | 1 | 1 | 252 | 1 | 1 | 30 |
| Control | 2 900 | 2 750 | -6,21 | 2 690 | 1 | 1 | 1 | 108 | 1 | 1 | 31 |
| Control | 2 940 | 2 650 | -7,14 | 2 580 | 1 | 1 | 1 | 240 | 4 | 2 | 35 |
| Control | 2 940 | 2 810 | -7,14 | 2 700 | 1 | 1 | 1 | 278 | 1 | 1 | 36 |
| CLF | 2 950 | 2 840 | -6,10 | 2 750 | 1 | 1 | 1 | 53 | 1 | 1 | 31 |
| Control | 2 950 | 2 860 | -6,10 | 2 720 | 2 | 3 | 3 | 173 | 1 | 1 | 34 |
| Control | 2 970 | 2 840 | -6,40 | 2 730 | 1 | 1 | 1 | 144 | 1 | 1 | 32 |
| CLF | 2 970 | 2 880 | -5,39 | 2 800 | 1 | 2 | 3 | 144 | 1 | 1 | 33 |
| Control | 2 970 | 2 780 | -6,40 | 2 700 | 3 | 3 | 3 | 279 | 1 | 1 | 33 |
| CLF | 2 980 | 2 860 | -5,70 | 2 800 | 1 | 1 | 2 | 181 | 1 | 1 | 35 |
| Control | 2 990 | 2 810 | -6,35 | 2 770 | 1 | 1 | 1 | 236 | 1 | 1 | 34 |
| Control | 3 050 | 2 980 | -5,25 | 2 890 | 1 | 3 | 3 | 144 | 1 | 1 | 35 |
| Control | 3 050 | 2 810 | -6,89 | 2 710 | 1 | 1 | 3 | 199 | 4 | 2 | 42 |
| CLF | 3 050 | 2 930 | -5,25 | 2 890 | 1 | 1 | 2 | 166 | 1 | 1 | 36 |
| CLF | 3 070 | 2 930 | -5,54 | 2 890 | 1 | 1 | 1 | 165 | 1 | 1 | 40 |
| CLF | 3 070 | 2 860 | -5,54 | 2 820 | 1 | 1 | 1 | 7 | 1 | 1 | 28 |
| CLF | 3 080 | 3 010 | -6,17 | 2 870 | 1 | 1 | 1 | 180 | 4 | 2 | 32 |
| Control | 3 080 | 2 850 | -7,79 | 2 800 | 1 | 3 | 3 | 38 | 3 | 2 | 30 |
| Control | 3 080 | 2 880 | -5,19 | 2 800 | 1 | 1 | 1 | 280 | 1 | 1 | 35 |
| Control | 3 100 | 2 910 | -5,48 | 2 890 | 1 | 2 | 2 | 30 | 1 | 1 | 32 |
| CLF | 3 110 | 2 980 | -6,75 | 2 900 | 1 | 1 | 1 | 160 | 3 | 2 | 29 |
| CLF | 3 110 | 2 930 | -5,79 | 2 880 | 1 | 1 | 1 | 130 | 4 | 2 | 36 |
| Control | 3 120 | 3 000 | -5,45 | 2 910 | 1 | 1 | 1 | 167 | 1 | 1 | 34 |
| Control | 3 120 | 2 850 | -6,09 | 2 730 | 1 | 1 | 2 | 222 | 1 | 1 | 25 |
| Control | 3 130 | 2 950 | -7,03 | 2 840 | 1 | 2 | 3 | 222 | 1 | 2 | 26 |
| CLF | 3 150 | 3 010 | -5,40 | 2 910 | 1 | 1 | 1 | 152 | 4 | 2 | 34 |
| Control | 3 160 | 2 960 | -5,06 | 2 890 | 1 | 2 | 3 | 157 | 4 | 2 | 35 |
| Control | 3 170 | 3 080 | -5,99 | 2 980 | 1 | 1 | 2 | 96 | 3 | 2 | 29 |
| Control | 3 180 | 3 120 | -5,97 | 2 910 | 1 | 1 | 3 | 120 | 1 | 1 | 33 |
| CLF | 3 190 | 3 260 | -5,02 | 3 030 | 1 | 1 | 1 | 33 | 1 | 1 | 30 |
| Control | 3 200 | 2 970 | -5,63 | 2 940 | 1 | 3 | 3 | 165 | 4 | 2 | 30 |
| CLF | 3 230 | 3 070 | -7,12 | 2 980 | 1 | 1 | 1 | 114 | 2 | 1 | 34 |
| CLF | 3 250 | 3 050 | -6,15 | 2 950 | 1 | 3 | 3 | 206 | 4 | 2 | 28 |
| Control | 3 260 | 3 090 | -6,13 | 2 960 | 1 | 1 | 1 | 255 | 1 | 1 | 34 |
| CLF | 3 280 | 3 140 | -6,10 | 3 050 | 1 | 1 | 1 | 163 | 1 | 1 | 29 |
| CLF | 3 290 | 3 180 | -5,78 | 3 070 | 1 | 1 | 1 | 69 | 4 | 2 | 38 |
| CLF | 3 300 | 3 200 | -5,45 | 3 070 | 1 | 1 | 2 | 171 | 1 | 1 | 31 |
| CLF | 3 310 | 3 070 | -5,74 | 3 050 | 1 | 1 | 1 | 165 | 4 | 2 | 32 |
| Control | 3 330 | 3 310 | -6,91 | 3 050 | 1 | 1 | 1 | 238 | 3 | 2 | 30 |
| Control | 3 360 | 3 120 | -5,95 | 3 030 | 1 | 1 | 1 | 193 | 4 | 2 | 21 |
| CLF | 3 370 | 3 200 | -5,93 | 3 130 | 1 | 2 | 3 | 117 | 3 | 2 | 30 |
| CLF | 3 370 | 3 230 | -6,53 | 3 150 | 1 | 1 | 1 | 141 | 1 | 1 | 30 |
| Control | 3 380 | 3 110 | -7,99 | 3 080 | 2 | 3 | 3 | 210 | 1 | 1 | 35 |
| CLF | 3 390 | 3 140 | -7,37 | 3 100 | 1 | 1 | 1 | 199 | 1 | 1 | 34 |
| Control | 3 400 | 3 220 | -5,00 | 3 150 | 1 | 1 | 1 | 75 | 1 | 1 | 33 |
| CLF | 3 410 | 3 430 | -5,28 | 3 230 | 1 | 1 | 1 | 179 | 3 | 2 | 26 |
| Control | 3 410 | 3 330 | -5,57 | 3 190 | 1 | 1 | 2 | 146 | 1 | 1 | 34 |
| Control | 3 410 | 3 270 | -5,57 | 3 220 | 1 | 3 | 3 | 137 | 1 | 1 | 30 |
| CLF | 3 410 | 3 170 | -7,33 | 3 150 | 1 | 1 | 1 | 168 | 1 | 2 | 32 |
| Control | 3 420 | 3 200 | -8,48 | 3 110 | 1 | 1 | 1 | 202 | 1 | 1 | 25 |
| CLF | 3 420 | 3 390 | -5,26 | 3 210 | 1 | 1 | 1 | 195 | 1 | 2 | 31 |
| CLF | 3 420 | 3 400 | -5,26 | 3 240 | 1 | 1 | 1 | 235 | 1 | 1 | 38 |
| CLF | 3 430 | 3 260 | -5,25 | 3 220 | 1 | 1 | 3 | 140 | 1 | 1 | 38 |
| Control | 3 440 | 3 230 | -8,14 | 3 130 | 1 | 1 | 1 | 132 | 1 | 1 | 24 |
| CLF | 3 450 | 3 210 | -8,12 | 3 170 | 1 | 1 | 1 | 173 | 4 | 2 | 34 |
| Control | 3 450 | 3 190 | -8,70 | 3 090 | 1 | 1 | 1 | 130 | 1 | 1 | 30 |
| Control | 3 470 | 3 180 | -8,36 | 3 090 | 2 | 3 | 3 | 201 | 3 | 2 | 32 |
| CLF | 3 490 | 3 310 | -5,16 | 3 280 | 1 | 1 | 1 | 236 | 1 | 1 | 29 |
| Control | 3 490 | 3 170 | -6,88 | 3 030 | 1 | 1 | 1 | 128 | 4 | 2 | 30 |
| CLF | 3 500 | 3 250 | -6,00 | 3 180 | 1 | 3 | 3 | 168 | 1 | 1 | 31 |
| CLF | 3 510 | 3 300 | -5,70 | 3 250 | 1 | 1 | 2 | 205 | 4 | 2 | 27 |
| CLF | 3 530 | 3 320 | -6,52 | 3 280 | 1 | 2 | 2 | 136 | 1 | 2 | 39 |
| Control | 3 540 | 3 470 | -5,37 | 3 340 | 1 | 1 | 1 | 254 | 1 | 1 | 36 |
| Control | 3 540 | 3 400 | -5,08 | 3 290 | 1 | 1 | 1 | 66 | 4 | 2 | 33 |
| Control | 3 540 | 3 280 | -8,76 | 3 200 | 1 | 1 | 1 | 132 | 3 | 1 | 30 |
| CLF | 3 550 | 3 260 | -9,30 | 3 080 | 1 | 1 | 1 | 118 | 4 | 2 | 29 |
| Control | 3 560 | 3 300 | -7,02 | 3 230 | 1 | 1 | 1 | 133 | 1 | 1 | 31 |
| CLF | 3 560 | 3 370 | -5,90 | 3 310 | 1 | 1 | 1 | 98 | 4 | 2 | 31 |
| Control | 3 580 | 3 230 | -7,26 | 3 210 | 1 | 1 | 2 | 238 | 1 | 1 | 31 |
| CLF | 3 600 | 3 320 | -5,83 | 3 270 | 1 | 1 | 3 | 239 | 3 | 2 | 37 |
| CLF | 3 600 | 3 380 | -7,22 | 3 330 | 1 | 1 | 1 | 171 | 1 | 1 | 27 |
| CLF | 3 610 | 3 350 | -9,70 | 3 250 | 1 | 1 | 1 | 141 | 4 | 2 | 30 |
| CLF | 3 620 | 3 470 | -6,08 | 3 380 | 1 | 3 | 3 | 97 | 3 | 2 | 25 |
| CLF | 3 620 | 3 390 | -7,18 | 3 340 | 1 | 1 | 1 | 144 | 1 | 1 | 30 |
| CLF | 3 630 | 3 500 | -5,23 | 3 380 | 1 | 2 | 3 | 213 | 1 | 1 | 25 |
| Control | 3 640 | 3 400 | -7,69 | 3 320 | 1 | 1 | 2 | 254 | 1 | 1 | 30 |
| Control | 3 650 | 3 520 | -5,48 | 3 450 | 1 | 2 | 3 | 172 | 1 | 1 | 36 |
| CLF | 3 650 | 3 500 | -8,22 | 3 340 | 1 | 1 | 2 | 176 | 1 | 1 | 28 |
| Control | 3 650 | 3 540 | -6,30 | 3 380 | 2 | 1 | 1 | 261 | 1 | 1 | 33 |
| CLF | 3 650 | 3 490 | -8,49 | 3 340 | 1 | 1 | 1 | 116 | 1 | 1 | 30 |
| Control | 3 660 | 3 490 | -5,19 | 3 420 | 1 | 3 | 3 | 166 | 1 | 1 | 29 |
| CLF | 3 660 | 3 420 | -5,19 | 3 400 | 1 | 1 | 2 | 198 | 1 | 1 | 30 |
| Control | 3 710 | 3 400 | -6,74 | 3 320 | 1 | 3 | 3 | 41 | 3 | 2 | 34 |
| CLF | 3 720 | 3 490 | -5,91 | 3 450 | 2 | 1 | 2 | 178 | 4 | 2 | 32 |
| Control | 3 720 | 3 400 | -5,38 | 3 330 | 1 | 3 | 3 | 209 | 4 | 2 | 30 |
| Control | 3 720 | 3 550 | -5,65 | 3 470 | 1 | 1 | 1 | 196 | 4 | 2 | 43 |
| Control | 3 760 | 3 650 | -5,05 | 3 540 | 1 | 1 | 2 | 140 | 1 | 1 | 30 |
| Control | 3 770 | 3 420 | -8,49 | 3 370 | 1 | 1 | 1 | 98 | 1 | 1 | 28 |
| CLF | 3 840 | 3 590 | -5,21 | 3 560 | 1 | 1 | 3 | 187 | 1 | 1 | 25 |
| Control | 3 860 | 3 930 | -5,70 | 3 610 | 1 | 1 | 1 | 104 | 4 | 2 | 33 |
| CLF | 3 890 | 3 700 | -5,40 | 3 560 | 1 | 1 | 1 | 138 | 1 | 1 | 33 |
| CLF | 3 920 | 3 830 | -5,36 | 3 710 | 1 | 1 | 1 | 106 | 1 | 1 | 36 |
| Control | 3 950 | 3 590 | -5,06 | 3 560 | 2 | 2 | 2 | 153 | 4 | 2 | 37 |
| CLF | 3 990 | 3 780 | -6,77 | 3 700 | 1 | 1 | 1 | 186 | 1 | 1 | 38 |

CLF=Controlled Limited Formula

Breastfeeding: 1=exclusive breastfeeding, 2=any breastfeeding, 3=formula only

Mode of delivery: 1=vaginal delivery, 2=assisted vaginal delivery (forceps or vacuum assisted), 3=acute cesarean section, 4=planned cesarean section

Skin-to-skin contact: 1=Yes, 2=No
