# Supplementary material for: Limited Amount of Formula May Facilitate Breastfeeding: Randomized, Controlled Trial to Compare Standard Clinical Practice versus Limited Supplemental Feeding
Source: PLoS One. 2016 Feb 26;11(2):e0150053. doi: 10.1371/journal.pone.0150053 (PMC4769147; doi:10.1371/journal.pone.0150053)
Supplement: S1 Text — (PDF) [file pone.0150053.s003.pdf]

Title:

- A prospective, randomized trial comparing the use of controlled limited formula (CLF) to standard approach (SA) with respect to efficiency and duration of breastfeeding in neonates. ELF study

Sponsor:

- Institute for the Care of Mother and Child, Prague

- Charles University, Prague - Research Project PRVOUK 32 (Institutional support for research at Charles University since 2012. The schemes are financed from the funding received from the state budget to support the University's long-term strategic development as a research organization known as 'institutional funding').

Principal investigator:

- Zbyněk Straňák, MD, PhD

Place:

- Institute for the Care of Mother and Child, Prague, Czech Republic

Protocol number:

- N/A – single centre study

Table of contents:

1.Introduction

1.1 Background

1.2 Purpose

2. Study objectives

2.1 Primary objective

2.2 Secondary objectives

3. Investigational plan

3.1 Study design

3.2 Rationale of study design

3.3 Rationale of dose/regimen, and duration of intervention

3.4 Rationale for choice of comparator

3.5 Risks and benefits

4. Population

4.1 Inclusion criteria

4.2 Exclusion criteria

5. Visit schedule and assessment

6. Safety monitoring

6.1 Adverse events

6.2 Serious adverse events

7. Consent

8. Statistical analysis

9. Case report form

1.Introduction

1.1. Background

Breastfeeding is considered to be the best and most cost-effective intervention in newborns in order to reduce morbidity, improve growth and short-term as well as long-term wellbeing. The properties of human breast milk help to facilitate developmental changes during critical periods of brain, immune system or gut development. Major organisations, including the World Health Organisation and UNICEF, recommend exclusive breastfeeding for the first 6 months and continued breastfeeding for 2 years or more with adequate complementary feeding, as longer duration of breastfeeding has been shown to associate with greater health benefits. For the newborn these include a reduction in the risk of common infectious and allergic diseases, as well as obesity, diabetes or sudden infant death syndrome. On the mothers' side, benefits of breastfeeding include reduction in the risk of type 2 diabetes, ovarian and breast cancer. Early cessation of breastfeeding in addition seems to be associated with a higher risk of maternal postpartum depression.

Although the benefits of breastfeeding are widely acknowledged, maintaining exclusive breastfeeding in longer-term remains to be a challenge. WHO states that globally less than 40% of infants under six months of age are exclusively breastfed. National Center for Chronic Disease Prevention and Health Promotion stated that in 2011 although 79% of newborn infants started to breastfeed, only 49% were breastfeeding at 6 months and 27% at 12 months of age. In Czech Republic (place of the study) the rate for exclusive breastfeeding is even lower (15.3%) and recent analysis revealed no differences in breastfeeding at discharge between Baby Friendly Initiative Hospitals and other hospitals.

To enable mothers to establish and sustain exclusive breastfeeding, WHO and UNICEF suggest initiation of breastfeeding within the first hour of life; breastfeeding without any additional food or drink, not even water; breastfeeding on demand as well as avoidance of bottles, teats and pacifiers. In general public health efforts today tend to emphasise reduction in the use of formula during the birth hospitalisation in order to improve breastfeeding rates and duration. Some published literature suggests additional formula feeds during birth hospitalisation may associate with earlier discontinuation of breastfeeding, others however have shown no benefit to duration of breastfeeding from formula restricting policy during birth hospitalisation.

A study focusing on the problems experienced by breastfeeding mothers shows that one of the most common reasons cited by mothers who opt to stop breastfeeding in early neonatal period

is anxiety over insufficient milk supply. The physiological process of onset of lactation does not usually occur immediately after delivery; instead it may take up to several days for mature milk production to settle in. It is the relatively small volumes of colostrum and the unsettled behaviour of the newborn prone to dissatisfaction during these first days of life that, despite offered reassurance, may be misinterpreted by the mother as insufficient milk supply and lead to early cessation of breastfeeding. Approaches to this problem vary widely. Although some, including The Baby Friendly Hospital Initiative promote elimination of supplementary top-up formula feeds for healthy infants, others perceive benefits from early supplementation of other fluids or foods. Optimizing clinical practice regarding establishing breastfeeding and formula use during early neonatal period may prove to have a major impact on overall health benefit for newborn infants, if this helps to prolong overall breastfeeding duration and raise the numbers of ideally exclusively breastfed infants up to 6 months of age.

1.2 Purpose of the study

In this study, we aim to investigate the role and effect of limited formula use on breastfeeding and its discontinuation. We hypothesize, that early limited formula feeds in infants with early weight loss will not adversely affect the rate of breastfeeding in short-term and long-term. Infants who lose 5% or more of their birth weight during the first 48 hours after delivery are known to be at higher risk of excessive weight loss and hence are at higher risk of eventual formula supplementation as well as the risk of maternal concern regarding insufficient milk supply with the feared result of shorter breastfeeding duration. These at risk newborns may hence benefit from an early limited formula use, where the given controlled amounts for a limited time would not be expected to interfere with breastfeeding as recommended.

2. Study objectives

2.1 Primary objective

- Exclusive breastfeeding at 3 months of age (value: Yes or Not)

2.2 Secondary objectives

- Breastfeeding at 3 months of age (value: Yes or No or to some extent)
- Breastfeeding at 6 months of age (value: Yes or No or to some extent)
- Exclusive breastfeeding at 6 months of age (value: Yes or Not)

- Weight loss - the loss of weight during birth hospitalization (calculated as actual weight/birth weight x 100)
- Maximal volume of supplemental feeds: summ of all supplemental feeds during hospitalization in ml)
- breastfeeding success-rate depending on the mode of delivery
- breastfeeding success-rate depending on the skin to skin contact at the delivery room

3. Investigational plan

3.1 Study design

- This study will be conducted as prospective, randomized, controlled and not blinded. The use of formula or breastfeeding makes it impossible to mask the study.
- Eligibility (Inclusion/Exclusion Criteria) will be checked by attending physician before a patient can be enrolled. Informed consent (ICF) will be obtained from the parent(s) or guardian(s) of all infants.
- Patients will be randomized 1:1 to one of the following 2 arms - controlled limited formula (CLF) group or to standard approach (SA) group.
- We will use the envelope technique for randomization (random permuted blocks, 13 blocks with 8 envelopes - 1:1).
- The randomization will be prepared independently by an administrative department.
- Allocation will be provided be nursing staff after obtaining ICF.
- The study coordinator collecting data at follow-up at 3 and 6 months (phone calls) remains blinded to group allocation.

3.2 Rationale of study design

A study design that randomly assigns participants into an experimental group = controlled limited formula group (CLF) or a control group = standard approach group (SA). The differences between the control and experimental groups will be studied. Planned number of participants: 52 patients in each group, experimental group and control group.

3.3 Rationale of dose/regimen, and duration of intervention

Infants in the CLF group (intervention group) will be given a set volume of 10ml of formula after each breastfeed until adequate milk production begins. The given dose of 10 ml of formula has been chosen as appropriate for newborns with birth weight 2500-4000 grams.

Duration of intervention: formula administration from enrolment (see inclusion criteria) until onset of lactation. All formula feeds will always be given using the syringe-technique.

Infants in the controlled limited formula group (CLF - intervention group) will be given a set volume of 10ml of formula (HIPP NE, HIPP Inc., Germany) after each breastfeed until adequate milk production begins.

HIPP NE is very well tolerated formula with low osmolality (236 mOsmol/l), based on colostrum (56 Kcal/l), contains LCP for the development of the brain and vision (twice as much LCP in the fat as mature breast milk) and hydrolysed whey protein in accordance with recommendation (ESPGAN). HIPP NE formula is easily digestible, free of sacharose, fructose and gluten.

3.4 Rationale for choice of comparator

Infants in the SA group (control group) will be exclusively breastfed. In the SA group supplemental feeds will be administered only in indicated cases (excessive weight loss more than 10%, irritability of the newborn and on mothers´ specific request). The type of supplemental feeding in control group is based on the mothers´ request (donor milk, formula).

3.5 Risks and benefits

Comparing the two groups will help to find out if giving controlled amounts of formula may help support mothers and their babies in establishing breastfeeding and keeping the rates of breastfeeding up in longer term (3 months and 6 months).

There are no known additional risks to participating as the comparison is with a standard approach to breastfeeding and formula feeding during hospitalisation after birth.

4. Population

4.1 Inclusion criteria

- Healthy term neonate (Gestational age between 37+1 – 41+6 weeks)
- Birth weight between 2 500 – 4 000 grams
- Absence of a severe neonatal/perinatal pathology
- Apgar score ≥ 8 at 5 minutes
- Uncomplicated delivery
- Normal postnatal adaptation
- Absence of severe congenital defects
- Healthy mother of Czech nationality
- Newborn from a singleton pregnancy
- Mother motivated to breastfeed for a long time
- body weight loss ≥ 5 per cent between 24th and 48th hour of life

4.2 Exclusion criteria

- Mothers producing no milk 24 – 48 hours after delivery
- Premature neonates
- Small and/or large for gestational age
- Serious maternal complications (hypertension, diabetes, systemic diseases, etc.)
- Mothers using therapy that might affect breastfeeding (i.e. antidepressants)
- Neonates with hypoglycaemia and/or neonates fed from birth for some reason
- Mother with an early formula introduction schedule (e.g. return to work)
- Drug abuse in mothers

5. Visit schedule and assessment

All healthy, term, inpatients born in the Institute for the care of mother and child will be checked for eligibility. If enrolment criteria are fulfilled and informed consent of parent(s)/guardian(s) is given, patient will be entered into the study and visited daily by the attending physician as per guidelines and standards of the hospital. All participating patients will be followed up at the age of 3 and 6 months per telephone interview. In case of any complications occurring after discharge from the hospital, the clinical status of the infant will be discussed with the appropriate general practitioner. All information and follow up results will be documented in detail in the specifically designed case report form.

6. Safety monitoring

6.1 Adverse events

Adverse Event (AE): any untoward medical occurrence in a patient or clinical trial subject administered a medicinal product and which does not necessarily have a causal relationship with the study intervention.

Therefore an Adverse Event can be any unfavourable and unintended sign (including an abnormal laboratory finding), symptom or disease temporally associated with the use of study intervention, whether or not considered related.

Adverse events of special interest:

- Vomiting
- Gastric residuals
- Feeding intolerance

- Abdominal distension
- Absence of stooling
- Failure to thrive

Adverse Reaction (AR): all untoward and unintended responses to the investigational medicinal intervention (controlled limited formula).
All adverse events judged by the Local Principal Investigator, Chief Investigator or the Sponsor as having a reasonable suspected causal relationship to the investigational intervention qualify as an **Adverse Reaction.**

6.2 Serious adverse events

Serious Adverse Event or Serious Adverse Reaction (SAE/R)“: any untoward medical occurrence or effect that at any dose results in:
1. death,
2. is life-threatening,
3. requires hospitalisation or prolongation of existing hospitalisation,
4. results in persistent or significant disability or incapacity,

Other important adverse events/reactions that may jeopardise the subjects or may require medical or surgical intervention to prevent one of the above 4 outcomes defining seriousness from occurring should also be considered serious. Such events could be:

- Overdose (accidental or intentional)
- An alarming adverse event/ experience
- Adverse events and/or laboratory abnormalities

Serious adverse events of special interest

- Necrotizing enterocolitis

SAE report: All events will be reported within 24 hours of occurance to the principal investigator and annualy to the local ethics committee. Phone: 603431682, mail.: z.stranak@seznam.cz

7. Consent

Informed consent will be obtained from the parent(s) or guardian(s) of all infants before randomization.

Ethical Committee Review

The trial protocol will be reviewed and approved by the appropriate ethical review committee (Local Ethics Committee). All trial personnel will implement the clinical trial with full respect and compliance of the legal and ethical European institutional requirements and codes of practices. Procedures involving newborn infants will conform to the Declaration of Helsinki (Seoul 2008) and the EMA guidelines on clinical research in neonates; guidance document 267484/2007.

8. Statistical analysis

A descriptive part of the data analysis will be based on the calculation of basic statistics (number of valid values, minimum, maxim, mean, and standard deviation for numeric variables; frequencies and percentages for categorised variables). Graphs will be used for graphical representation (histogram or box plot for numeric variables; pie chart or column chart for categorised variables).

For comparison of groups of categorised variables, the analysis will be based on the pivot table (frequencies, row percentages, Chi-Square Test of independence, or Fisher's Exact Test).

To show comparisons of groups of numeric variables, tables with descriptive statistics in the groups, or box plot for the groups or panel histogram will be used. The Mann-Whitney test will be performed to compare the groups.

The calculations will be made using the software IBM SPSS Statistics.

9.Case report form

Case Report Form

Patient ID number:

The patient fulfils all inclusion criteria for the study:

Yes No

The patient fulfils any exclusion criteria of the study:

Yes No

Informed consent has been signed by the parent/ guardian:

Yes No

Demographic data

Maternal age:

Maternal education: University degree College graduate High school Elementary school

I plan to breastfeed for at least 3 months: Yes No

Number of deliveries:

Duration of breastfeeding after previous pregnancy:

Previously encountered problems with breastfeedingí: Yes No

Source of obtained information about breastfeeding:

- Internet Friends Maternity unit other

Newborn:

Birth weight (g):

Lenght at birth (cm):

Head circumference (cm):

Thorax circumference (cm):

Gestational age (completed weeks+days):

Type of delivery:

Vaginal Assisted vaginal delivery Acute caesarean section Planned caesarean section

Skin to skin contact in delivery room: Yes No

Time of enrollment (between 24-48 hours):

Randomization

INTERVENTION GROUP **CONTROL GROUP**

1st day

Frequency of breastfeeding during first day after randomization:

Weight on first day after randomization:

Weight 1: Weight 2:

Form of supplemental feeding in intervention group:

- Alternative (syringe) Bottle Other

Any supplemental feeds administered in control group?: Yes No

Requested form of supplemental feeds in control group:

Breastmilk from the breastmilk-bank own expressed breastmilk Formula

Form of supplemental feeding in control group:

- Alternative (syringe) Bottle Other

2nd day

Frequency of breastfeeding during second day after randomization:

Total amount of breastmilk within 24 hours (ml):

Weight on second day after randomization:

Weight 1: Weight 2:

Form of supplemental feeding in intervention group:

- Alternative (syringe) Bottle Other

Any supplemental feeds administered in control group?: Yes No

Requested form of supplemental feeds in control group:

breastmilk from the breastmilk-bank own expressed breastmilk Formula

Form of supplemental feeding in control group:

- Alternative (syringe) Bottle Other

3rd day

Frequency of breastfeeding during third day after randomization:

Total amount of breastmilk within 24 hours (ml):

Weight on third day after randomization:

Weight 1: Weight 2:

Form of supplemental feeding in intervention group:

- Alternative (syringe) Bottle Other

Any supplemental feeds administered in control group?: Yes No

Requested form of supplemental feeds in control group:

breastmilk from the breastmilk-bank own expressed breastmilk Formula

Form of supplemental feeding in control group:

- Alternative (syringe) Bottle Other

4th day

Frequency of breastfeeding during fourth day after randomization:

Total amount of breastmilk within 24 hours (ml):

Weight on fourth day after randomization:

Weight 1: Weight 2:

Form of supplemental feeding in intervention group:

- Alternative (syringe) Bottle Other

Any supplemental feeds administered in control group?: Yes No

Requested form of supplemental feeds in control group:

breastmilk from the breastmilk-bank own expressed breastmilk Formula

Form of supplemental feeding in control group:

- Alternative (syringe) Bottle Other

5th day

Frequency of breastfeeding during fifth day after randomization:

Total amount of breastmilk within 24 hours (ml):

Weight on fifth day after randomization:

Weight 1: Weight 2:

Form of supplemental feeding in intervention group:

- Alternative (syringe) Bottle Other

Any supplemental feeds administered in control group?: Yes No

Requested form of supplemental feeds in control group:

breastmilk from the breastmilk-bank   own expressed breastmilk   Formula

Form of supplemental feeding in control group:

- Alternative (syringe)   Bottle   Other

6th day

Frequency of breastfeeding during sixth day after randomization:

Total amount of breastmilk within 24 hours (ml):

Weight on sixth day after randomization:

Weight 1:   Weight 2:

Form of supplemental feeding in intervention group:

- Alternative (syringe)   Bottle   Other

Any supplemental feeds administered in control group?:   Yes   No

Requested form of supplemental feeds in control group:

breastmilk from the breastmilk-bank   own expressed breastmilk   Formula

Form of supplemental feeding in control group:

- Alternative (syringe)   Bottle   Other

ADVERSE EVENTS SECTION

Feeding disorders:

Yes   No

- Regurgitation of amniotic fluid: Yes   No
- Vomiting: Yes   No
- Sucking insufficiency: Yes   No
- Inability to breastfeed: maternal factors   newborn factors
- Breastfeeding aids used: Yes   No

Feeding intolerance: Yes   No

- Abdominal distension: Yes   No
- Delayed meconium (more than 48 hours):   Yes   No
- Gastric residuals: Yes   No

More than 10% weight loss: Yes   No

Necrotizing enterocolirtis: Yes   No

FOLLOW UP SECTION

Breastfeeding at time of discharge:

Exclusively Partially Formula only

Weight at time of discharge:

At 3 moths of age:

Exclusive breastfeeding Partial breastfeeding Formula only

Overall health status: good altered (medical intervention)

Reasons for medical intervention:

At 6 moths of age:

Exclusive breastfeeding Partial breastfeeding Formula only

Overall health status: good altered (medical intervention)

Reasons for medical intervention:

Failure to thrive: Yes No

OTHER COMPLICATIONS OF SPECIAL INTEREST

Highest value of transcutaneous icterometry on sternum:

Highest level of serum bilirubin (if measured)

Phototherapy: Yes No

Duration of phototherapy (hours):

Hypoglycemia: Yes No Not applicable

Proven birth defects: Yes No

Neuro-muscular disorders (e.g. decreased muscle tone, hyperreflexia): Yes No

Sepsis: Yes No

Serious maternal complications with negative effect on breastfeeding: Yes No

Other:
