# Supplementary material for: Limited Amount of Formula May Facilitate Breastfeeding: Randomized, Controlled Trial to Compare Standard Clinical Practice versus Limited Supplemental Feeding
Source: PLoS One. 2016 Feb 26;11(2):e0150053. doi: 10.1371/journal.pone.0150053 (PMC4769147; doi:10.1371/journal.pone.0150053)
Supplement: S2 Text — (Original document in Czech language.) (PDF) [file pone.0150053.s004.pdf]

# Case Report Form

**Rodné číslo pacienta:**

**Pacient splňuje kritéria pro zařazení do studie:**

Ano Ne

**Pacient splňuje kritéria pro nezařazení do studie:**

Ano Ne

**Zákonný zástupce podepsal informovaný souhlas:**

Ano Ne

**Demografická data**

Věk matky:

Nejvyšší dosažené vzdělání matka: VŠ SŠ s maturitou SŠ ZŠ

Mám v plánu kojit alespoň 3 měsíce: Ano Ne

Počet porodů:

Délka kojení u předchozího dítěte:

Obtíže při kojení u předchozích dětí: Ano Ne

**Informační zdroje o kojení:**

- Internet Rady kamarádek Rady v porodnici jiné

**Dítě:**

Porodní hmotnost (g):

Porodní délka (cm):

Obvod hlavy (cm):

Obvod hrudníku (cm):

Gestační stáří (týden+den):

**Způsob porodu:**

Vaginální Vaginální operativní Akutní per S.C. Plánovaný per S.C.

**Přiložení na PS:** Ano Ne

**Čas zařazení do studie (24-48 hodin):**

**Randomizace**

**INTERVENCE NON-INTERVENCE**

**1.den**

Frekvence kojení 1. den po zařazení:

Hmotnost 1. den po zařazení:

Hmotnost 1: Hmotnost 2:

**Způsob aplikace formule u intervenční skupiny:**

- Alternativně Savička Jiné

**Dokrm u non-intervenční skupiny:** Ano Ne

**Požadovaný druh výživy u non-intervenční skupiny:** ŽML OMM Formule

Způsob aplikace formule u non - intervenční skupiny:

- Alternativně Savička Jiné

**2.den**

Frekvence kojení 2. den po zařazení:

Celkové množství MM/24 hodin (ml):

Hmotnost 2. den po zařazení:

Hmotnost 1: Hmotnost 2:

Způsob aplikace formule u intervenční skupiny:

- Alternativně Savička Jiné

**Dokrm u non-intervenční skupiny:** Ano Ne

**Požadovaný druh výživy u non-intervenční skupiny:** ŽML OMM Formule

Způsob aplikace formule u non - intervenční skupiny:

- Alternativně Savička Jiné

**3.den**

Frekvence kojení 3. den po zařazení:

Celkové množství MM/24 hodin (ml):

Hmotnost 3. den po zařazení:

Hmotnost 1: Hmotnost 2:

Způsob aplikace formule u intervenční skupiny:

- Alternativně Savička Jiné

**Dokrm u non-intervenční skupiny:** Ano Ne

**Požadovaný druh výživy u non-intervenční skupiny:** ŽML OMM Formule

Způsob aplikace formule u non - intervenční skupiny:

- Alternativně Savička Jiné

**4.den**

Frekvence kojení 4. den po zařazení:

Celkové množství MM/24 hodin (ml):

Hmotnost 4. den po zařazení:

Hmotnost 1: Hmotnost 2:

Způsob aplikace formule u intervenční skupiny:

- Alternativně Savička Jiné

**Dokrm u non-intervenční skupiny:** Ano Ne

**Požadovaný druh výživy u non-intervenční skupiny:** ŽML OMM Formule

Způsob aplikace formule u non - intervenční skupiny:

- Alternativně Savička Jiné

**5.den**

Frekvence kojení 5. den po zařazení:

Celkové množství MM/24 hodin (ml):

Hmotnost 5. den po zařazení:

Hmotnost 1: Hmotnost 2:

Způsob aplikace formule u intervenční skupiny:

- Alternativně Savička Jiné

**Dokrm u non-intervenční skupiny:** Ano Ne

**Požadovaný druh výživy u non-intervenční skupiny:** ŽML OMM Formule

Způsob aplikace formule u non - intervenční skupiny:

- Alternativně Savička Jiné

**6.den**

Frekvence kojení 6. den po zařazení:

Celkové množství MM/24 hodin (ml):

Hmotnost 6. den po zařazení:

Hmotnost 1: Hmotnost 2:

Způsob aplikace formule u intervenční skupiny:

- Alternativně Savička Jiné

**Dokrm u non-intervenční skupiny:** Ano Ne

**Požadovaný druh výživy u non-intervenční skupiny:** ŽML OMM Formule

Způsob aplikace formule u non - intervenční skupiny:

- Alternativně Savička Jiné

**Poruchy příjmu potravy:**

Ano Ne

- Ublinkávání VP: Ano Ne
- Zvracení: Ano Ne
- Špatné sání: Ano Ne
- Obtížné přiložení: mateřské faktory novorozenecké faktory
- Používání pomůcek ke kojení: Ano Ne

**Poruchy tolerance stravy:**

Ano Ne

- Distenze břicha: Ano Ne

- Opožděný odchod smolky (více než 48 hodin): Ano Ne
- Gastrická rezidua: Ano Ne

**Hmotnostní úbytek více než 10%:** Ano Ne

**Kojení při propuštění:**

Plně částečně nekojeno

Hmotnost při propuštění:

**KOMPLIKACE**

Maximální hodnoty TKI na hrudníku:

Maximální sérové hodnoty bilirubinu (pokud bylo měření provedeno):

Fototerapie: Ano Ne

Délka fototerapie (celkem hodin):

Hypoglykemie: Ano Ne N/A

Prokázaná VVV po narození: Ano Ne

Zjištěné neuro-svalové onemocnění (např. hypotonie, hyperreflexie, tonusová porucha): Ano Ne

Sepse: Ano Ne

Závažné komplikace u matky negativně ovlivňující kojení: Ano Ne

Jiné:

**Follow up:**

**Ve 3 měsících:**

Plně kojené částečně kojené nekojené

Celkový zdravotní stav: zdravé nemocné (návštěva lékaře a/nebo podávání léků)

Důvody návštěvy lékaře/medikace

**V 6 měsících:**

Plně kojené částečně kojené nekojené

Celkový zdravotní stav: zdravé nemocné (návštěva lékaře a/nebo podávání léků)

Důvody návštěvy lékaře/medikace
